# Bispecific antibodies come to the aid of cancer immunotherapy

**DOI:** 10.1002/1878-0261.12977

**Published:** 2021-05-14

**Authors:** Ivano Amelio, Gerry Melino, Arnold J. Levine

**Affiliations:** ^1^ Department of Experimental Medicine University of Rome Tor Vergata TOR Rome Italy; ^2^ School of Life Sciences University of Nottingham Nottingham UK; ^3^ Institute for Advanced Study Simons Center for Systems Biology Princeton NJ USA

## Abstract

Three collaborative studies published by the groups of Vogelstein, Gabelli, and Zhou report the development of specially designed bispecific antibodies that may help in overcoming the limitations of current immunotherapies. The bispecific antibodies have been designed to couple cells harboring HLA‐presented tumor‐specific antigens from Tp53 mutant or Ras mutant with CD4 and CD8 T cells, thus facilitating immune‐mediated clearance of the cancer cells.

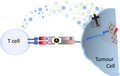

**Abbreviations**
HLA: human leukocyte antigen
ICI: immune checkpoint inhibitors
MHC: major histocompatibility complex locus
scDb: single‐chain diabody
scFv: single‐chain variable fragment
TAAs: tumor‐associated antigens
TSAs: tumor‐specific antigens

The use of immune checkpoint inhibitors (ICI) to activate adaptive immune responses against some tumor types has clearly been a major advance in cancer therapeutics [1]. Despite the significant impact of ICI implementation on the outcomes of cancers, treatments with ICI currently fail to eradicate the majority of tumors as a result of inadequate priming of an autologous tumor‐specific immune response. Three collaborative studies published by the groups of B. Vogelstein, S. B. Gabelli, and S. Zhou indicate that specially designed bispecific antibodies may help overcome limitations of ICI.

For an adaptive immune response to develop, intracellular proteins are processed by proteolysis into peptides. Antigenic peptides are then loaded onto transporters that move the peptide antigen to a receptor, which will present the antigen on the cell surface. These receptors are genetically encoded by a chromosome 6 region of highly repetitive DNA sequences, termed the major histocompatibility complex locus (MHC). In the human population, the MHC locus contains a large number of polymorphic genes encoding human leukocyte antigen (HLA) class I and class II molecules. Mutated peptides (neo‐peptides)—commonly derived from genetic missense mutations that accumulate during the process of tumor transformation—are loaded onto HLA class I and class II receptors and presented on the surface of cancer cells [2]. Tumor antigens are commonly classified as tumor‐associated antigens (TAAs, such as alpha‐fetoprotein) and tumor‐specific antigens (TSAs, such as neoantigens). The former are aberrantly expressed at high levels by cancer cells, but are also expressed by a subset of normal cells; hence, they are not absolutely specific to the cancer tissues. Conversely, TSAs are specifically found only in cancer cells, as they generally derive from gene products with somatic mutations in the coding region of the gene.

Priming autologous CD4 and CD8 T‐cell responses against tumors may fail due to several reasons: loss of cell surface HLA receptors; lack of TSAs, especially in tumors with a low tumor mutational burden; establishment of an immunosuppressive tumor microenvironment through the presence of myeloid‐derived suppressor cells; CD8 T‐cell exhaustion; and impaired co‐localization and interaction of CD4 T cells, CD8 T cells, and antigen‐presenting cells [2, 3, 4, 5].

Aiming at boosting tumor‐specific immune responses by improving immune cell co‐localization and interaction, B. Vogelstein, S. B. Gabelli, S. Zhou, and colleagues have developed bispecific antibodies that couple cells harboring HLA‐presented TSAs from a Tp53 mutant allele (R175H; Hsiue *et al*. [6]) or a Ras mutant (G12V, and Q61H, Q61R, or Q61L; Douglass *et al*. [7]) with CD4 and CD8 T cells. This work has been expanded in a third research project by the same teams, where the authors employed a similar concept to treat T‐cell malignancies with bispecific antibodies designed to bring together malignant T cells expressing a particular clonal TCR beta‐antigen with nonmalignant helper CD4 and killer CD8 T cells (Paul *et al*. [8]). These studies demonstrate that improved immune cell co‐localization and their interaction with tumor antigen‐presenting cells are required for the selective immune‐mediated killing of tumors.

The specially designed bispecific antibodies constituted from one antibody receptor that binds with high affinity to the mutant peptide–HLA complex on cancer cells, but not to its wild‐type counterpart on normal cells, and one antibody receptor that binds with the T‐cell receptor–CD3 complex on T cells. This antibody construct could successfully couple, in the same locality of the tumor or draining lymph nodes, the TSA presentation complex with T cells, leading to T‐cell activation and clonal expansion. Subsequently, TSA‐specific T cells were shown to secrete cytokines that indirectly aid cancer cell killing, while CD8 T cells directly attacked the tumor (Fig. 1). The Tp53 and Ras mutations targeted by these bispecific antibodies are among the most common missense mutations specific to cancer cells and, thereby, constitute excellent targets for priming tumor‐specific immune responses. However, only selected polymorphic HLA class I and class II receptors bind to Tp53 R175H or K‐Ras G12V mutant peptides [9] and they do so with a poor affinity, limiting the applicability of treatment with p53 R175H or K‐Ras G12V‐targeting bispecific antibodies to a subset of patients. Nevertheless, the authors have focused their bispecific antibodies upon the HLA‐A1 or HLA‐A3 series of receptors and have engineered antibody affinities so that even low levels of antigens can be detected.

**Fig. 1:**
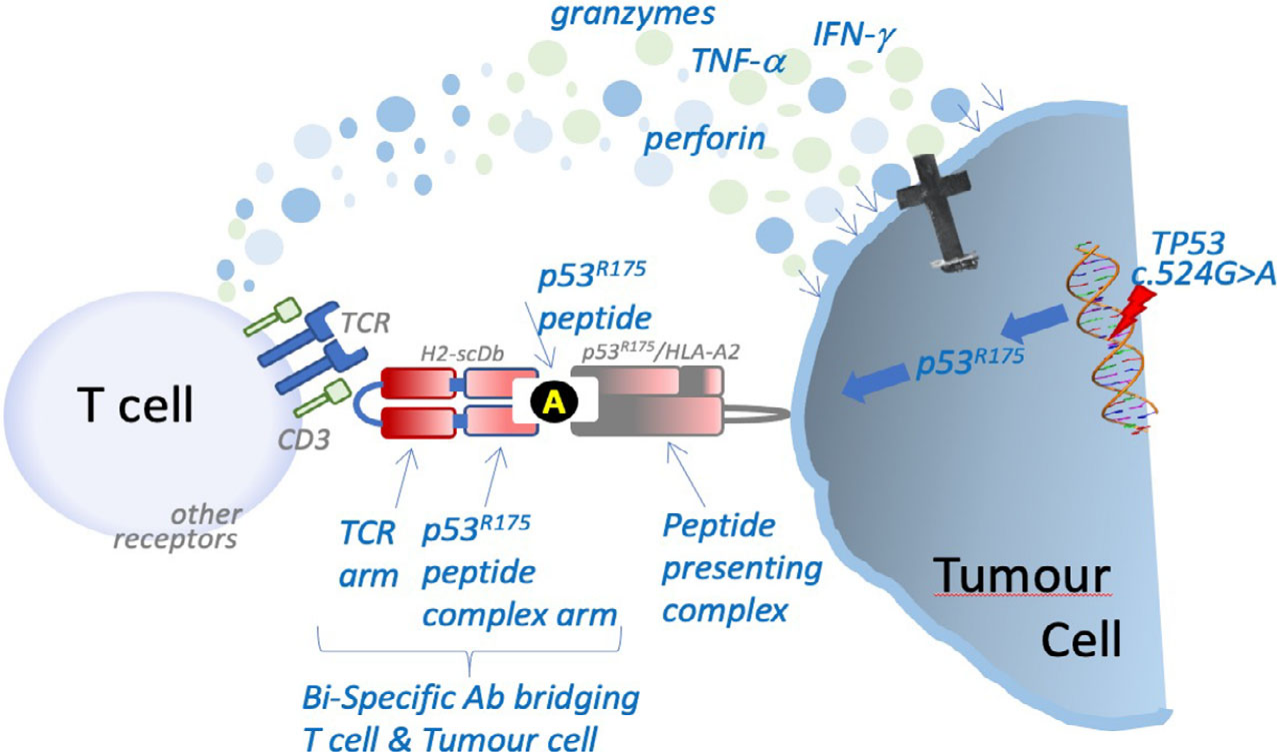
Schematic representation of the mechanism of action of single‐chain diabodies against p53 R175H. Antigenic peptides including the R175H mutant epitope of p53 are exposed on the surface of the cancer cells bound to the HLA complex. A scDb such as H2‐scDB, binding *via* its one arm to the antigen‐presenting HLA complex on the cancer cell and *via* its other arm to the CD3/TCR complex on T cells, thereby bringing T cells in close proximity with tumor antigen‐specific tumor cells. Subsequent T‐cell activation results in killing of the cancer cell, mediated through the release of cytotoxic molecules. CD3, cluster of differentiation 3; TCR, T‐cell receptor; TNF‐α, tumor necrosis factor‐alpha; IFN‐γ, interferon‐gamma.

To target the neoantigen derived from the *TP53* mutation R175H (arginine‐to‐histidine 175 substitution)^10^, ^11^, Hsiue *et al*. conducted a phage display positive selection of naïve human antibody libraries against HLA‐A*02:01 peptide–HLA monomers that contained the p53 R175H peptide, combined with a negative selection against peptide–HLA monomers containing the p53 wild‐type peptide. The selected phage clones were then converted into a bispecific antibody, linking in a single‐chain diabody (scDb) format each individual single‐chain variable fragment (scFv) to an anti‐CD3e scFv (UCHT1), which confers the ability to bind CD3 and activates a polyclonal T‐cell response. Specificity of the top ranked phage peptide binder scDbs (including H2‐scDb) for p53 R175H was further proven using functional assays assessing specific binding to cells expressing p53R175H. Importantly, scDb did neither bind to cells expressing wild‐type p53 proteins, nor bind to cell lines expressing other p53 missense mutations and cell lines where p53 R175H expression was deleted using CRISPR‐mediated genome editing. These results indicated low risk of cross‐reactivity and off‐target effects. X‐ray crystallography studies shed light onto the structural basis of scDb binding to mutated p53R175H/HLA‐A*02:01, further supporting target selectivity. H2‐scDb selectively killed p53 R175H cancer cells *in vitro* and *in vivo* in a polyfunctional T‐cell‐dependent manner. No treatment effect was observed following administration of H2‐scDb in NOD‐SCID‐Il2rg^−/−^ mice engrafted with p53 R175H, but in the absence of combination engraftment with human T cells [6].

An analogous strategy and experimental validation process were employed to develop scDbs targeting HLA alleles conjugated to RAS G12V and RAS Q61H, RAS Q61L, or RAS Q61R. Douglass *et al*. [7] identified scDbs displaying strong selectivity and high affinity against cancer cells expressing low levels of specific RAS mutants. Cross‐reactivity to other human peptides of similar or related composition cannot yet be excluded. After all both p53 and *Ras* belong to gene families (*p53, p63, and p73* [12, 13, 14, 15, 16] and *H‐ras, K‐ras, and N‐ras* [17]) that are expressed widely in diverse cell types. Hopefully, the high affinity reported for the bispecific antibody will also translate into high specificity when the bispecific antibodies are administered to patients.

A slightly different strategy was followed to develop scDbs against T‐cell malignancies. Targeting T‐cell cancers with antibodies that kill all healthy and malignant T cells is not a viable therapeutic option as healthy T cells are required for a functioning immune system. Hence, therapeutic antibodies need to eradicate malignant T cells while sparing the majority of nonmalignant T cells. The TCR β‐chain variable regions can be composed through the rearrangement of 30 possible *TRBV* family members, and in clonal T‐cell malignancies, only a single TRBV region is expressed on the surface of the malignant T cells, while polyclonal nonmalignant T cells express a range of different TCR β‐family members. Thus, TCR b‐chain expressed by malignant T cells represents an ideal TAA for therapeutic applications. As proof‐of‐concept, Paul *et al*. developed scDbs against TRBV5‐5 or TRBV12, tethered to a CD3‐specific antibody. These agents could selectively eliminate malignant human T cells transplanted into mice, preserving the majority of healthy human T cells, which did not express the targeted TRBV. The authors did not select the targeting antibody with a phage display experimental procedure but employed previously identified TRBV5‐5 and TRBV12‐specific antibodies [8]. Of note, bispecific antibodies have previously been used to develop and target aberrantly expressed, nonmutated, cancer proteins (TAAs)^18^.

Collectively, these publications demonstrate that targeting TSAs (mutated oncogenes/oncosuppressors) with bispecific antibodies is a reasonable approach to cancer therapy in humans. TSAs presented in the context of a specific HLA type have been targeted previously by *in vitro* T‐cell expansion and transfer back into patients [19]. A disadvantage of that approach, compared with the use of scDbs, is that adaptive cell therapies require a time‐consuming and expensive set of manipulations of patient‐derived immune cells. The use of scDbs could conversely represent an ‘off‐the‐shelf’ pre‐prepared easily accessible and, thus, widely applicable therapeutic set of reagents.

The significance of the studies by B. Vogelstein, S. B. Gabelli, S. Zhou, and colleagues lies within the formal and high‐quality demonstrations of the feasibility of bispecific antibody design that ensures the high affinity that is required for targeting and eliminating cancer cells with good specificity (recognizing between mutant *vs* wild‐type TSAs in the HLA pocket). The ability to achieve such a high affinity and specificity could switch the emphasis of cancer immunotherapy approaches from some cell‐based strategies to clinical implementation of bispecific antibodies. Nevertheless, the route to taking these bispecific antibodies into the clinic is long, as several issues remain to be evaluated. Previous trials with some bispecific antibodies have triggered an overexpression of innate immune system cytokines (such as IL‐6) resulting in toxic shock or organ failures [2]. Other responses to bispecific antibodies that liberate transforming growth factor‐beta and IL‐10 can give rise to CD4 regulatory (Treg) cells that suppress the immune system instead of activating it [2]. The immune system is well known to change with the age of the patient, or past environmental history, and is sexually dimorphic. One may well expect to obtain different responses to the manipulation of the immune system based upon age, sex, or past exposures to the microbiome or chemical or physical interventions [20, 21, 22, 23, 24]. Many tumors deploy an immunosuppressive repertoire of signaling molecules, and how scDbs will function in this landscape is an open question. Indeed, mutations in the p53 pathway themselves, activated by *Ras* mutations, alter the innate immune responses of some cells (senescence‐activated secretory proteins) changing the expected immune activities [2]. The pairing of K‐ras and Tp53 mutations in some cancers is quite common because a *Ras* mutation activates p53 activity. It would be of some interest for the authors to explore the treatment of these cancers using both bispecific antibodies to target K‐ras and Tp53 mutations simultaneously.

In spite of these difficulties, we need to learn how to arm the immune system so that it truly eliminates tumors or, even better, prevents tumors from arising. Until then, it seems unlikely that newly developed small molecules or drugs will efficiently eliminate tumors in the absence of a tumor‐specific immune response [25]. So, in spite of the long list of difficulties in testing immune‐altering reagents in humans we must go on to obtain a deeper understanding of the complexities of the homeostatic immune system of humans.

## Conflict of interest

The authors declare no conflict of interest.
